# Cushing’s Disease: Subclinical Left Ventricular Systolic and Diastolic Dysfunction Revealed by Speckle Tracking Echocardiography and Tissue Doppler Imaging

**DOI:** 10.3389/fendo.2017.00222

**Published:** 2017-09-05

**Authors:** Beata Uziębło-Życzkowska, Paweł Krzesinński, Przemysław Witek, Grzegorz Zielinński, Agnieszka Jurek, Grzegorz Gielerak, Andrzej Skrobowski

**Affiliations:** ^1^Department of Cardiology and Internal Diseases, Military Institute of Medicine, Warsaw, Poland; ^2^Department of Endocrinology and Isotope Therapy, Military Institute of Medicine, Warsaw, Poland; ^3^Department of Neurosurgery, Military Institute of Medicine, Warsaw, Poland

**Keywords:** Cushing’s disease, global longitudinal strain, hypertension, cardiac systolic and diastolic dysfunction

## Abstract

**Objective:**

Novel echocardiographic techniques, such as speckle tracking echocardiography (STE) and tissue Doppler imaging, are sensitive tools for assessing left ventricular (LV) performance. LV global longitudinal strain (GLS), assessed by STE, is a sensitive marker of myocardial systolic function. Cardiovascular complications in patients with Cushing’s disease (CD) determine a higher mortality than that in an age- and gender-matched population. Cardiac systolic dysfunction may be detected in early stages by STE. Thus, the aim of this study was to investigate the usefulness of STE in detecting subclinical LV dysfunction in three groups of patients: CD group, arterial hypertension group (AHG), and healthy volunteers (HV).

**Methods:**

Echocardiographic assessments of LV systolic and diastolic function were performed in 171 subjects (CD: 22, AHG: 114, HV: 35) with no symptoms of heart failure. A statistical comparison included separate analyses for men and women.

**Results:**

CD patients showed good blood pressure (BP) control (below 140/90 mmHg in 82% of cases). However, in comparison AHG and HV groups they exhibited: (1) significantly lower LV contractility expressed by GLS (CD group: −17.7%, AHG group: −19.2%, HV: −20.0%; *p* = 0.004) and (2) higher prevalence of LV diastolic dysfunction (45.0, 14.2, 0.0%, respectively; *p* < 0.00001). Men with CD showed significantly more pronounced LV diastolic dysfunction. Cortisol excess in women was related to impaired LV systolic function.

**Conclusion:**

CD, even with well-controlled BP, is associated with LV dysfunction which depends individually on sex. These hemodynamic alterations can be detected by modern non-invasive diagnostic tools and may become potential therapeutic objectives.

## Introduction

Cushing’s disease (CD) is a severe endocrine disease resulting in a chronic excess of glucocorticoids (1). CD is associated with a twofold to fivefold increase in mortality compared with that in the general population, mainly due to cardiovascular complications (2, 3). Glucose homeostasis alterations (32% with diabetes and 27% with pre-diabetes) are typical for patients with CD. Arterial hypertension (AH) is the most common cardiovascular disease in patients with CD. The available data shows that about 70–85% of adult patients and 50–78% of pediatric patients with CD suffer from AH (4). AH is very common for all types of hypercortisolism (both endogenous and exogenous). It seems that hypertensive patients with CD should be treated like other hypertensive patients. The authors said that angiotensin-converting enzyme (ACE) inhibitors and/or angiotensin receptor blockers (ARBs) are recommended antihypertensive medications in CD patients (5). Calcium antagonists and/or mineralocorticoid receptor antagonists are also acceptable in more severe AH. One should bear in mind that clinical control of hypertension could be difficult in the presence of hypercortisolism. Moreover, patients with CD have a higher risk of other cardiovascular diseases. Patients with an active form of CD have higher rates of heart failure and acute myocardial infarction than healthy people (4). CD patients have also been reported to exhibit higher rates of structural left ventricular (LV) alterations in the form of LV hypertrophy and concentric remodeling as well as functional LV alterations, such as decreased LV systolic performance and impaired diastolic filling (5).

Conventional echocardiography has some limitations in the evaluation of LV performance. Left ventricular ejection fraction (LVEF) is usually normal in patients with isolated AH as well as in patients with CD. Speckle tracking echocardiography (STE), a modern, well-validated, and reproducible method of assessing LV longitudinal deformation, offers a more sensitive assessment of myocardial contractility. Evaluating global longitudinal strain (GLS) may help identify patients with subclinical LV systolic dysfunction.

The aim of this study was to investigate the usefulness of STE in detecting subclinical LV systolic and diastolic dysfunction in three groups of subjects: CD patients, essential hypertension patients, and healthy volunteers (HV).

## Materials and Methods

### Study Population

A comparative retrospective analysis was performed in the following groups of age-matched subjects:

- 22 consecutive patients with CD qualified for transsphenoidal surgery, including 73% with a history of treated AH but no other cardiovascular diseases (CD group, CD),
- 114 patients with essential AH without other concomitant diseases [arterial hypertension group (AHG)],
- 35 HV, non-obese, non-smoking, with normal blood pressure (BP) (HV group, HV).

The AH group comprised patients with at least 3-month history of AH defined according to the 2013 European Society of Cardiology (ESC) guidelines (6), enrolled in clinical study NCT01996085 (ClinicalTrials.gov). Exclusion criteria for the AH group were: (1) secondary AH, (2) AH treated with three or more medicines before recruitment, (3) heart failure, (4) cardiomyopathy, (5) severe heart rhythm disorders, (6) significant valvular disease, (7) kidney failure (GFR below 60 mL/min*1.73 m^2^), (8) chronic obstructive pulmonary disease, (9) diabetes, (10) polyneuropathy, (11) peripheral vascular disease, (12) age <18 years and >65 years, and (13) body mass index (BMI) >40 kg/m^2^.

The CD group consisted of patients with Cushing’s disease diagnosed based on standard hormonal criteria: increased urinary free cortisol (UFC), increased serum cortisol levels at 8.00 a.m., loss of cortisol circadian rhythm, increased or detectable plasma adrenocorticotropic hormone (ACTH) levels at 8.00 a.m., and failure to suppress serum cortisol levels to <1.8 mg/dL during an overnight dexamethasone suppression test (1 mg of dexamethasone administered at midnight). The pituitary etiology of Cushing’s syndrome was confirmed based on serum cortisol or UFC suppression >50% with a high-dose dexamethasone suppression test (2 mg q6h for 48 h), positive corticotrophin-releasing hormone stimulation test (100 μg i.v.), and positive pituitary MRI findings. Inferior petrosal sinus sampling was performed in all cases of microadenomas smaller than 6 mm, equivocal MRI findings, or inconsistent dynamic test results.

Glycemic status data were recorded in the CD group, with the patients qualified as diabetic, pre-diabetic, or individuals with normal glucose tolerance. In all patients with CD, the standard hormonal assessment of gonadotroph and thyrotroph function was performed.

It should be emphasized that the evaluated CD patients took no medications affecting the pituitary–adrenal axis function, and the pituitary thyrotroph function was either preserved (21 cases) or well-controlled with l-thyroxin therapy (1 case). Therefore, neither steroidogenesis inhibitors nor estrogen therapy nor pituitary deficiency interfered with echocardiography results. The transsphenoidal surgery was curative in 19 out of 22 patients with CD (86.4%). All 19 patients in remission and one in three patients with persistent CD had positive ACTH immunohistochemistry.

The studies were conducted according to Good Clinical Practice guidelines and the Declaration of Helsinki. The study protocol had been approved by the Ethics Committee of the Military Institute of Medicine in Warsaw. Written informed consents were obtained from all the patients.

### Clinical Examination

The clinical examination focused mainly on cardiovascular risk factors, such as family history of cardiovascular disease or nicotinism, and BMI. Office BP measurements were automated (Omron M4 Plus, Japan) according to ESC guidelines (6). Office systolic blood pressure (SBP) and office diastolic blood pressure (DBP) measurements were performed in the presence of a trained physician or nurse, in a quiet room, in a sitting position after a minimum of 5 min of rest, with the arm supported and legs uncrossed. Subjects were instructed not to smoke; drink alcohol, coffee, or other fluids potentially increasing BP; or take drugs potentially affecting BP (e.g., pain killers) for a minimum 12-h period before the measurement. The mean of two measurements of both SBP and DBP was included in the final analysis.

### Echocardiography

Standard parasternal, apical, and subcostal views were used in two-dimensional echocardiography (2.5 MHz transducer; VIVID S6 GE Medical System, Wauwatosa, WI, USA). The parasternal long-axis view was used to measure the left atrial (LA) diameter, left ventricular end diastolic diameter (LVEDD), and interventricular septum thickness at end-diastole (IVSd). The LVEF was calculated according to Simpson’s method.

The apical 4-chamber view with the pulsed-wave Doppler gate positioned in the LV at the tips of mitral valve leaflets was used to calculate mitral valve inflow: mitral flow early (E) and late (A) phase ratio (E/A), and phase E deceleration time. Simultaneous registration of the flow pattern through the aortic and mitral valves and the calculation of isovolumic diastolic time were performed in the apical 5-chamber view. Mitral annular velocity was measured in apical views by tissue Doppler imaging (TDI) with the sample volume positioned at or 1 cm within the septal insertion site of the mitral leaflets to cover the longitudinal excursion of the mitral annulus in diastole. Moreover, the early diastolic velocity (e′) of the septal mitral annulus was measured to calculate the E/e′ ratio. Left ventricular diastolic dysfunction (LVDD) was diagnosed according to current guidelines (7).

Left ventricular GLS was assessed with the use of automated function imaging software. The analysis involved ECG-gated digital images in the apical long-axis 2-chamber and apical 4-chamber views. High temporal resolution of >50 frames per second was obtained to assure acoustic-marker tracking. With two basal points selected at the level of the mitral annulus and the third point at the apex detection of the tracked area was carried out semi-automatically, with the possibility of manual adjustments. LV walls were divided into six segments in each apical view, and tracking quality and the value of strain were assessed for each LV segment. The mean global longitudinal peak systolic strain was calculated for each view. The mean of these values was the value of GLS. Statistical analyses were performed for the entire study population and for men and women separately.

### Statistical Analysis

Statistical analyses were performed using Statistica 12.0 (StatSoft, Inc., Tulsa, OK, USA). Data distribution and normality were assessed by visual inspection and the Kolmogorov–Smirnov test. Continuous variables were presented as means ± SD and categorical variables as absolute and relative frequencies (percentages). To analyze the differences between subgroups the ANOVA/Kruskal–Wallis test was used for continuous variables and the chi-square test/Fisher exact test for categorical variables. A *p*-value of <0.05 was adopted to indicate statistical significance.

## Results

### Baseline Characteristics

Basic characteristic of three groups was presented in Table 1. The CD group showed good BP control and the mean systolic/diastolic BP was 123/81 mmHg (82% of subjects had BP below 140/90 mmHg). Only slight differences in basic clinical parameters were observed (HR, SBP, DBP, BMI). There were no differences in left ventricular mass index (LVMI) values, left atrial, or LV dimensions.

**Table 1:** Inter-group comparison for the whole group.

	Arterial hypertension group (AHG) *n* = 114	Cushing’s disease (CD) *n* = 22	Healthy volunteers (HV) *n* = 35	*p*-Value	*Post hoc* tests
Age (years), mean ± SD	46.3 ± 9.3	42.9 ± 12.5	43.8 ± 9.1	0.180	–
HR (bpm), mean ± SD	68.1 ± 8.7	73.9 ± 10.3	66.6 ± 11.7	0.016	AHG vs HV*
					HV vs CD*
Systolic blood pressure (mmHg), mean ± SD	120.7 ± 9.5	123.2 ± 13.8	114.5 ± 10.1	0.002	AHGvs HV**
					HV vs CD**
Diastolic blood pressure (mmHg), mean ± SD	77.9 ± 7.3	80.8 ± 9.7	72.3 ± 8.0	0.0001	AHG vs HV^#^
					HV vs CD^#^
Body mass index (kg/m^2^), mean ± SD	30.9 ± 21.7	31.8 ± 7.1	25.8 ± 3.2	0.301	–
Echocardiography					
LVH, *n* (%)	17 (14.9)	9 (40.9)	3 (8.6)	0.002	
LVDD, *n* (%)	16 (14.0)	9 (40.9)	0 (0.0)	0.00005	
LVEDD (mm), mean ± SD	48.4 ± 3.8	47.2 ± 4.0	48.4 ± 4.2	0.430	–
RVEDD (mm), mean ± SD	30.5 ± 3.2	30.4 ± 4.3	28.7 ± 4.0	0.036	AHG vs HV*
LA (mm), mean ± SD	37.3 ± 3.4	36.5 ± 3.9	35.0 ± 3.2	0.003	AHG vs HV**
Left ventricular mass index (g/m^2^), mean ± SD	90.1 ± 18.0	101.9 ± 22.7	83.4 ± 20.1	0.004	AHG vs CD*
					HV vs CD**
LVEF (%), mean ± SD	66.4 ± 3.2	66.9 ± 3.3	67.5 ± 3.5	0.256	–
GLS (%), mean ± SD	−19.2 ± 2.4	−17.7 ± 2.0	−20.0 ± 2.3	0.004	AHG vs CD*
					HV vs CD**
E/A (–), mean ± SD	1.15 ± 0.34	1.00 ± 0.28	1.25 ± 0.33	0.025	HV vs CD*
E′ (cm/s), mean ± SD	10.4 ± 2.6	9.7 ± 3.7	12.6 ± 2.6	0.00006	AHGvs HV^#^
E/e′, mean ± SD	7.0 ± 1.9	7.2 ± 1.7	5.9 ± 1.2	0.003	HV vs CD^#^

*p < 0.05; **p < 0.01; ^#^p < 0.001.

*E/A, mitral flow early (E) and late (A) phase ratio; E/e′, mitral flow early (E) phase and mitral septal annulus early diastolic velocity (e′) ratio; GLS, global longitudinal strain; LA, left atrium diameter; LVDD, left ventricular diastolic dysfunction; LVEDD, left ventricular end diastolic diameter; LVEF, left ventricular ejection fraction; LVH, left ventricular hypertrophy; RVEDD, right ventricular end diastolic diameter*.

Seventeen patients (77.3%) in the CD group and all patients (100%) in the AH group has been treated AH. None of them has taken more than three antihypertensive medications. Most of the patients have taken 1 or 2 medications. AH group patients had at least 3 months’ history of antihypertensive treatment but the exact duration of antihypertensive therapy in the AH and CD groups is unknown. Diabetes was confirmed in 7 out of 22 CD patients (32%), pre-diabetes in 6 patients (27%), whereas 9 patients (41%) had normal glucose tolerance. In the group of patients with diabetes five were treated with metformin only and two with metformin and insulin.

Twenty-one out of 22 CD patients had preserved anterior pituitary function. One patient with an invasive corticotroph tumor had been diagnosed with thyrotropin deficiency, but it was well-controlled on a stable dose of l-thyroxin.

### Echocardiographic Findings

**Figure 1:**
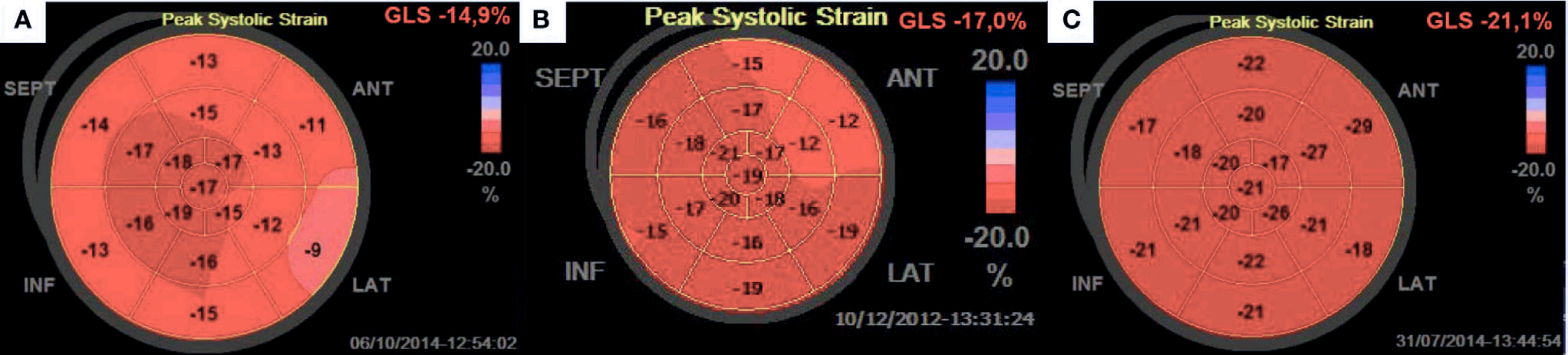
Bullseye strain recording in **(A)** Cushing’s disease; **(B)** arterial hypertension, and **(C)** a healthy volunteer.

In comparison with the AHG and HV groups, the CD group exhibited:

(1) significantly lower LV contractility expressed by GLS (CD group −17.7%, AHG group −19.2%, HV −20.0%; *p* = 0.004) despite comparable LVEF (66.9, 66.4, 67.5%, respectively; ns) (Figure 1; Table 1).
(2) higher prevalence of LV diastolic dysfunction (40.9, 14.0, 0.0%; *p* < 0.00001) related to a lower E/A ratio (1.00, 1.15, 1.25; *p* = 0.025); lower e′ value (9.7, 10.4, 12.6 cm/s; *p* = 0.00006), and higher E/e′ ratio (7.21, 7.02, 5.88; *p* = 0.003) (Table 1).

Men with CD distinguished with worse both LV diastolic and systolic function (GLS: −17.2%, −18.8%, −19.6%; *p* = 0.052; and the e′ value: 8.3, 10.5, 12.6 cm/s; *p* = 0.0002) (Table 2). Hypercortisolism in women was more related to an impaired LV systolic function (GLS: −18.0, −20.0, −21.1%; *p* = 0.010; and e′: 10.5, 10.0, 12.5 cm/s; ns) than in men (Table 3).

**Table 2:** Inter-group comparison for men.

	Arterial hypertension group (AHG) *n* = 76	Cushing’s disease (CD) *n* = 8	Healthy volunteers (HV) *n* = 24	*p*-Value	*Post hoc* tests
Age (years), mean ± SD	45.3 ± 9.3	45.0 ± 13.4	41.8 ± 7.7	0.276	–
HR (bpm), mean ± SD	68.7 ± 9.0	71.3 ± 10.5	65.3 ± 11.4	0.223	–
Systolic blood pressure (mmHg), mean ± SD	121.6 ± 9.6	132.1 ± 12.9	116.6 ± 9.4	0.0007	AHG vs CD*
					HV vs CD^#^
Diastolic blood pressure (mmHg), mean ± SD	78.3 ± 7.3	84.3 ± 10.1	73.3 ± 8.0	0.001	AHG vs HV*
					HV vs CD**
Body mass index (kg/m^2^), mean ± SD	29.1 ± 3.6	34.1 ± 8.1	26.2 ± 2.4	<0.00001	AHGvs HV**
					AHG vs CD**
					HV vs CD^#^
Echocardiography					
LVH, *n* (%)	8 (10.5)	3 (37.5)	3 (12.5)	0.055	–
LVDD, *n* (%)	11 (14.5)	4 (50.0)	0 (0.0)	0.008	–
LVEDD (mm), mean ± SD	49.5 ± 3.1	48.6 ± 2.9	49.8 ± 3.8	0.685	–
RVEDD (mm), mean ± SD	31.4 ± 2.8	33.4 ± 2.2	30.0 ± 3.0	0.016	HV vs CD*
LA (mm), mean ± SD	38.6 ± 2.6	38.3 ± 3.3	36.8 ± 3.2	0.0004	AHG vs HV*
Left ventricular mass index (g/m^2^), mean ± SD	91.8 ± 16.5	111.8 ± 20.2	89.0 ± 20.9	0.012	AHG vs CD*
					HV vs CD*
LVEF (%), mean ± SD	66.1 ± 3.5	66.6 ± 3.6	67.3 ± 3.3	0.328	–
GLS (%), mean ± SD	−18.8 ± 2.2	−17.2 ± 2.1	−19.6 ± 2.2	0.001	AHG vs CD**
					HV vs CD**
E/A (–), mean ± SD	1.18 ± 0.35	0.84 ± 0.20	1.30 ± 0.35	0.008	AHG vs CD*
					HV vs CD**
E′ (cm/s), mean ± SD	10.5 ± 2.7	8.3 ± 2.9	12.6 ± 2.4	0.0002	AHG vs HV**
					HV vs CD^#^
E/e′, mean ± SD	6.6 ± 1.6	7.4 ± 1.9	5.9 ± 1.1	0.0495	HV vs CD*

*p < 0.05; **p < 0.01; ^#^p < 0.001.

*E/A, mitral flow early (E) and late (A) phase ratio; E/e′, mitral flow early (E) phase and mitral septal annulus early diastolic velocity (e′) ratio; GLS, global longitudinal strain; LA, left atrium diameter; LVDD, left ventricular diastolic dysfunction; LVEDD, left ventricular end diastolic diameter; LVEF, left ventricular ejection fraction; LVH, left ventricular hypertrophy; RVEDD, right ventricular end diastolic diameter*.

**Table 3:** Inter-group comparison for women.

	Arterial hypertension group (AHG) *n* = 38	Cushing’s disease (CD) *n* = 14	Healthy volunteers (HV) *n* = 11	*p*-Value	*Post hoc* tests
Age (years), mean ± SD	48.2 ± 9.0	41.6 ± 12.3	48.0 ± 10.9	0.112	–
HR (bpm), mean ± SD	66.9 ± 7.4	78.8 ± 9.2	70.2 ± 7.8	0.021	AHG vs CD*
Systolic blood pressure (mmHg), mean ± SD	119.1 ± 9.1	118.1 ± 11.9	110.2 ± 10.5	0.040	AHG vs HV*
Diastolic blood pressure (mmHg), mean ± SD	77.2 ± 7.4	78.8 ± 9.2	70.2 ± 7.7	0.019	AHG vs HV*
					HV vs CD*
Body mass index (kg/m^2^), mean ± SD	34.3 ± 37.1	30.6 ± 6.4	24.9 ± 4.5	0.636	–
Echocardiography					
LVH, *n* (%)	9 (23.7)	6 (64.3)	0 (0.0)	0.038	
LVDD, *n* (%)	5 (13.2)	5 (35.7)	0 (0.0)	0.032	–
LVEDD (mm), mean ± SD	46.3 ± 4.1	46.5 ± 4.4	45.2 ± 3.3	0.680	–
RVEDD (mm), mean ± SD	28.9 ± 3.3	28.8 ± 4.3	25.8 ± 4.6	0.063	–
LA (mm), mean ± SD	34.8 ± 3.5	34.8 ± 3.6	33.2 ± 2.6	0.390	–
Left ventricular mass index (g/m^2^), mean ± SD	86.6 ± 10.5	96.5 ± 22.9	71.1 ± 10.8	0.013	HV vs CD**
LVEF (%), mean ± SD	67.0 ± 2.6	67.1 ± 3.3	67.8 ± 4.2	0.766	–
GLS (%), mean ± SD	−20.0 ± 2.5	−18.0 ± 2.0	−21.1 ± 2.7	0.010	AHG vs CD*
					HV vs CD*
E/A (–), mean ± SD	1.10 ± 0.31	1.08 ± 0.29	1.15 ± 0.29	0.851	–
E′ (cm/s), mean ± SD	10.0 ± 2.3	10.5 ± 2.9	12.5 ± 3.2	0.059	–
E/e′, mean ± SD	7.9 ± 2.0	7.1 ± 1.6	6.0 ± 1.5	0.014	AHG vs HV*

*p < 0.05; **p < 0.01.

*E/A, mitral flow early (E) and late (A) phase ratio; E/e′, mitral flow early (E) phase and mitral septal annulus early diastolic velocity (e′) ratio; GLS, global longitudinal strain; LA, left atrium diameter, LVDD, left ventricular diastolic dysfunction; LVEDD, left ventricular end diastolic diameter; LVEF, left ventricular ejection fraction; LVH, left ventricular hypertrophy; RVEDD, right ventricular end diastolic diameter*.

## Discussion

Our results revealed that patients with CD exhibited a more pronounced LV systolic and diastolic dysfunction than hypertensive patients and healthy individuals, with similar basic clinical presentation. These observations are clinically important as cardiovascular complications of cortisol excess strongly affect the prognosis in patients with CD. Population studies and clinical observations suggest that CD is associated with a higher mortality in comparison to that in general population. Death in CD is typically due to myocardial infarction or stroke, especially in patients receiving inadequate treatment (8–11). Therefore, identifying subclinical cardiac dysfunction before any clinical symptoms emerge is important for early initiation of cardioprotective treatment.

Myocardial strain is a measure of quantifying LV function, which is now feasible with STE. GLS is the most commonly evaluated strain parameter, reported to be a more sensitive measure of systolic function than LVEF (12). Thus, GLS assessments may be used to identify subclinical LV dysfunction at early stages of many diseases. GLS is a sensitive indicator of myocardial dysfunction due to factors, such as ischemia (13, 14), hypertrophy (15), hypoxia (16), cardiotoxic drugs (17), and several systemic illnesses (18). Furthermore, recent studies have shown that GLS abnormalities are independently associated with adverse cardiovascular outcomes (15, 19).

Results of our study revealed that patients with CD have significantly lower LV contractility expressed by GLS, even compared to patients with AH. Our previous results proved that GLS may be impaired despite a normal LVEF even at early stages of AH (20). Moreover, GLS correlated with some indices of LV diastolic function (e′, E/e′ ratio) (20). In the study presented here, patients with CD were characterized by a significant discordance between GLS and LVEF, even stronger than that in AH patients. Their LV systolic depression coexisted with LV diastolic dysfunction. Thus, we confirmed that GLS is a valuable biomarker of subclinical LV dysfunction in CD and is more sensitive than LVEF. GLS assessment *via* STE seems to be a novelty in diagnosing cardiovascular complications in CD. A recent study (21) has shown that patients with CD have impaired diastolic and systolic LV function (measured by TDI). Toja et al. (22) assessed LV hypertrophy and found that CD patients had higher LVMI than both normotensive and matched hypertensive controls. However, to the best of our knowledge, this is the first study reporting the use of STE in CD.

Chronically increased cardiac load seems to be the main cause of accelerated LV dysfunction. About 70–85% of adults with hypercortisolism (23, 24) suffer from hypertension and the duration of elevated blood cortisol levels seems to be correlated with the development of AH (23), the latter being an independent predictor of mortality in patients with CD (25). Increased arterial stiffness may play the crucial role. Bayram et al. (26) observed that aortic strain was significantly decreased in patients with CD compared with those in the control group. However, elevated BP is not the only factor that may lead to cardiac damage in CD. Myocardial fibrosis is an important ultrastructural abnormality directly related to the effects of cortisol, independent from AH (27). Yiu et al. (28) demonstrated that myocardial remodeling is significantly increased in untreated CD patients compared with that in patients with essential AH. This may explain, to some extent, the more impaired GLS in patients with AH caused by CD than in those with essential AH. As mentioned above, treatment of hypertensive patients with CD is difficult due to hypercortisolism. These patients usually need more intensive therapy. Moreover, hypertensive patients with CD had a higher risk of cardiovascular disease, even in low-grade HA. Therefore, in view of our findings, patients with subclinical diastolic and/or systolic cardiac dysfunction and borderline AH should be considered for treatment with ACE inhibitors or ARBs. These medications are known to have cardioprotective effects and an early treatment may be beneficial for these patients. Moreover, if STE shows systolic and/or diastolic subclinical cardiac dysfunction in hypertensive patients with CD, the therapy can be changed (e.g., ACE inhibitors or ARBs instead of calcium blockers or other antihypertensive medications).

A more detailed analysis of our results suggested that men with CD had a more impaired cardiac function than matched hypertensives and healthy individuals. Both LV systolic and diastolic dysfunction rates were higher in CD males, whereas impaired LV systolic function was only characteristic for females. Gender-related differences in patients with CD were also reported by other authors (29), who revealed that compared with women, men with CD were more prone to: osteoporosis, hypokalemia, sexual dysfunction, and hypertension (*p* < 0.05), had significantly higher preoperative and postoperative (6 months after surgery) cortisol levels (*p* < 0.001, *p* = 0.003) and a higher recurrence rate (*p* = 0.028). The clinical value of these observations should be further investigated. It is possible that young and middle-aged men with CD demand special and careful long-term follow-up.

### Clinical Implications

Our results confirm that subclinical heart disease is present in CD, even with well-controlled BP. Thus, the issue of early preventive pharmacotherapy emerges. Patients with CD and symptomatic heart disease are usually treated with standard guideline-based therapy. However, there is no sufficient evidence to give reliable therapeutic recommendations for those with asymptomatic LV dysfunction. By the time LVEF decline is detected it may be too late for effective treatment and complete restoration of LV function. Early cardioprotection may offer some benefits. We have recently noticed (30) that 12 months of effective anti- hypertensive treatment in asymptomatic hypertensive patients restored both LV systolic function (expressed by GLS) and LV diastolic function (expressed by E/A ratio, e′, and E/e′ ratio). In fact, those with the most explicit impairment of GLS at baseline improved the most in the end.

Assessing subclinical LV dysfunction by STE and TDI helps identify patients who might benefit the most from appropriate cardiac disease prevention. Moreover, detection of impaired cardiac function may prompt a more intensive therapeutic approach in patients previously treated with cardiovascular drugs. In view of our previous observations (20) impaired GLS is associated with higher afterload (systemic vascular resistance index), and its reduction may be an additional goal of antihypertensive therapy. Regular clinical and echocardiographic screening should include early signs of heart failure that demand specific therapeutic approach.

### Limitations

We realize that the small sample size and a retrospective design are limitations of this study. Moreover, we performed no diagnostic assessments, such as exercise test and/or coronary angiography, in order to exclude asymptomatic ischemic heart disease. However, no signs or symptoms suggesting cardiac ischemia, such as LV wall motion abnormalities, characteristic electrocardiographic findings, chest pain and/or dyspnea, were noted. Our study mainly involved young and middle-aged patients and our results should not be extrapolated to the general population. On the other hand, the strength of our results lay in the fact that we recruited subjects with no other serious chronic diseases apart from CD and AH. Thus, our observations were specific to these populations and deprived of potential bias related to additional alterations of cardiovascular function. Our patients with AH (of the CD and essential AH groups) were treated according to current recommendations (6). Our previous study (30) demonstrated no evidence that the administered medical treatment had any significant influence on GLS. The current study was not aimed at verifying the effects of different medications on the evaluated echocardiographic parameters. Thus, although our previous analysis showed no inter-drug differences, we cannot exclude diverse effects of different medications on LV performance. Finally, the duration of hypertension, which was not investigated in detail, might potentially confound the results.

## Conclusion

Cushing’s disease, even with well-controlled BP, is associated with LV systolic and diastolic dysfunction, whose character and/or severity depends individually on sex. These hemodynamic abnormalities can be detected by modern non-invasive diagnostic tools and may become potential therapeutic objectives.

## Ethics Statement

The studies were conducted according to Good Clinical Practice guidelines and the Declaration of Helsinki. The study protocol had been approved by the Ethics Committee of the Military Institute of Medicine in Warsaw. Written informed consents were obtained from all the patients.

## Author Contributions

Conception and design of the work and data acquisition, interpretation of the data, and revision of manuscript: BU-Ż, PK, PW, GZ, AJ, GG, and AS. Data analysis and drafting of manuscript: BU-Ż and PK. All the authors approved the final version.

## Conflict of Interest Statement

The authors declare that the research was conducted in the absence of any commercial or financial relationships that could be construed as a potential conflict of interest.
